# δ-Opioid receptor activation attenuates oxidative injury in the ischemic rat brain

**DOI:** 10.1186/1741-7007-7-55

**Published:** 2009-08-26

**Authors:** Yilin Yang, Xiwei Xia, Yi Zhang, Qiang Wang, Lu Li, Guanghua Luo, Ying Xia

**Affiliations:** 1 Third Clinical College of Schoow University, Changzhou, Jiangsu, PR China; 2 Yale University School of Medicine, New Haven, Connecticut, USA

## Abstract

**Background:**

We have recently shown that δ-opioid receptors (DORs) play an important role in neuroprotection from hypoxic injury via the regulation of extracellular signaling-regulated kinase (ERK) and cytochrome *c *release. Since ERK and cytochrome *c *are differentially involved in caspase signaling of oxidative injury that significantly contributes to neuronal damage in ischemia/reperfusion, we considered if DOR activation protects the ischemic brain by attenuating oxidative injury.

**Results:**

We observed that, in a model of cerebral ischemia with middle cerebral artery occlusion, DOR activation increased the activity of major antioxidant enzymes, glutathione peroxidase and superoxide dismutase, and decreased malondialdehyde and nitric oxide levels in the cortex exposed to cerebral ischemia/reperfusion. In addition, DOR activation reduced caspase 3 expression, though it did not significantly affect the increase in interleukin (IL)1β and tumor necrosis factor (TNF)α expression at the same timepoint. PD98059, an inhibitor of mitogen-activated protein kinase (MAPK) extracellular signaling-regulated kinase kinase, accelerated animal death during ischemia/reperfusion.

**Conclusion:**

DOR activation attenuates oxidative injury in the brain exposed to ischemia/reperfusion by enhancing antioxidant ability and inhibiting caspase activity, which provides novel insights into the mechanism of DOR neuroprotection.

## Background

Stroke induces hypoxic injury in the brain and is a leading cause of neurological disability and death in the world. Although prevention and early treatment of this condition are critical to reduce the devastating effects on individuals and their families, to date there has been no effective strategy to protect the brain from ischemic injury. Exploring the complex mechanisms of hypoxic/ischemic injury and finding new approaches against such injury have been a long-term battle and attracted much attention from both scientists and clinicians [1,2].

Our initial work found that activation of δ-opioid receptors (DORs) are protective against hypoxic/excitotoxic injury in cortical neurons [3-5]. Furthermore, we observed that DORs are involved in neuroprotection against hypoxic/ischemic insults in various models including neurons under hypoxia, brain slices exposed to hypoxia or oxygen-glucose deprivation and *in vivo *brain with cerebral ischemia [6-13]. More recently, substantial data generated from other independent laboratories has demonstrated that DORs are indeed neuroprotective against hypoxic or ischemic stress, either in *in vitro *neurons or *in vivo *models of brain or spinal ischemia [14-23]. For example, a recent study [18] elucidated the effect of [D-Ala^2^, D-Leu^5^]-enkephalinamide (DADLE, a DOR agonist) on hippocampal neurons in ischemia and found that intraperitoneal injection of DADLE improved hippocampal neuronal survival in Sprague-Dawley rats. In fact, DORs may be tonically involved in neuroprotection [4,22] through a Gi-dependent manner [22].

However, the mechanism underlying the DOR neuroprotection is not well understood. In an *in vitro *exploration, we found that DORs upregulate the activity of extracellular signaling-regulated kinase (ERK) and reduce the release of cytochrome *c*, thus protecting neurons from hypoxic injury [6]. Since ERK and cytochrome *c *are differentially involved in caspase signaling of oxidative injury that significantly contributes to ischemia/reperfusion (I/R) injury [24-26], we considered in this work the question of whether DOR activation can enhance antioxidant ability and thus attenuate oxidative injury in the brain exposed to I/R stress. For the purpose of comparison, we also determined whether DADLE affects the expression of some inflammatory cytokines such as interleukin (IL)1β and tumor necrosis factor (TNF)α, which are known to play a role in I/R injury [25-27].

## Results

**Figure 1:**
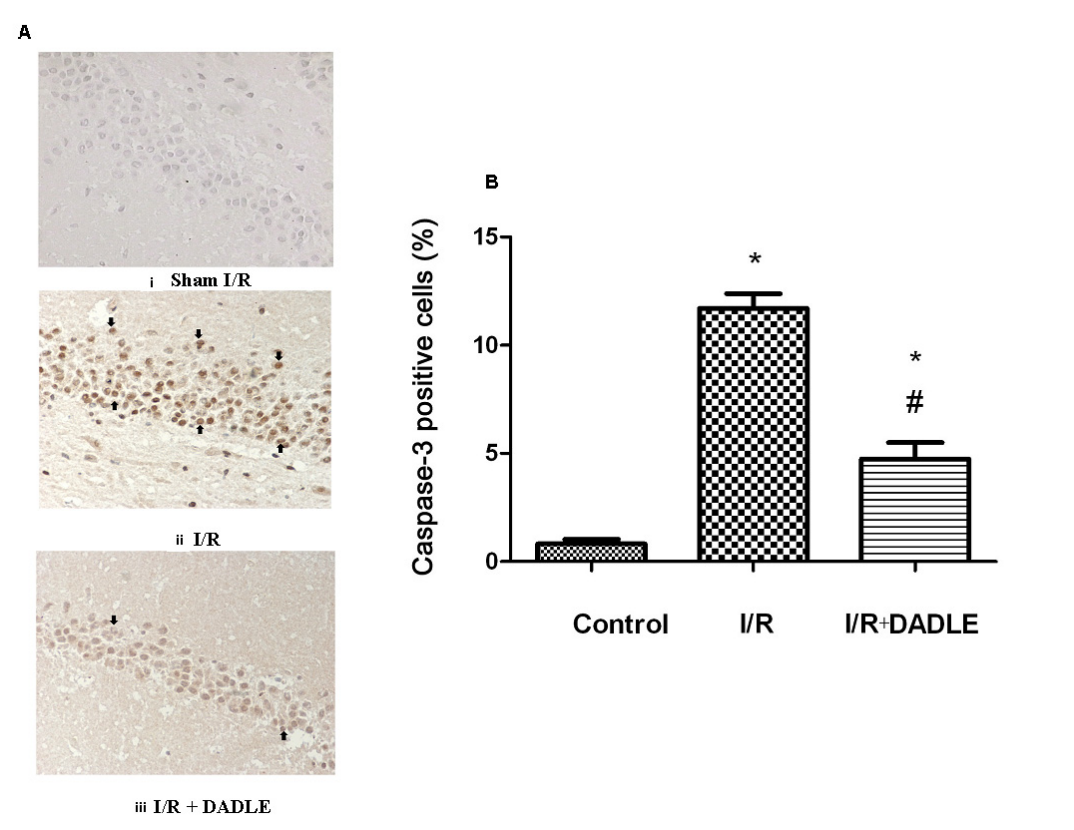
**[D-Ala^2^, D-Leu^5^]-Enkephalinamide (DADLE)-induced reduction of caspase 3 staining in the hippocampus exposed to ischemia/reperfusion (I/R) stress**. A, Representative images. Strept actividin-biotin complex (SABC), 10 × 40. (i) Sham I/R. (ii) I/R. (iii) I/R plus. Arrows indicate caspase 3-positive cells. Note that there was little caspase 3 staining in the sham control slice, while there was strong signal staining in that of I/R group, which was largely reduced by **δ**-opioid receptor (DOR) activation with DADLE. B, Quantification of caspase 3-positive cells. Mean ± standard error (n = 6). *, *P *< 0.05, I/R or I/R + DADLE versus control. #, *P *< 0.05, I/R + DADLE versus I/R. Note that the administration of DADLE reduced the number of caspase 3-positive cells in the hippocampus following the cerebral ischemia/reperfusion.

Our previous work and those of others have clearly demonstrated that DOR activation with DADLE or other DOR agonists attenuates hypoxic/ischemic injury in cortical neurons and reduces ischemic infarction in the cortex [3-23]. Since caspase activity plays a key role in apoptotic signal transduction and is a crucial indicator of ischemic injury [25,28,29], we studied whether DOR activation reduced caspase activity in the ischemic brain by examining caspase 3 expression using immunohistochemical methods. To mimic cerebral I/R injury, the model of stroke was established by performing MCAO in the rat as described in the methods. Since all the cortices were sampled for biochemical measurements, immunohistochemistry was performed in the hippocampi of the same animals. Unsurprisingly, we observed that DOR activation with DADLE reduced the expression of caspase 3 induced by MCAO. As shown in Figure 1, for example, there was little caspase 3 signal in the sham control slices, while there were more caspase 3-positive cells in the I/R group, which could be largely reduced by DOR activation with DADLE.

**Figure 2:**
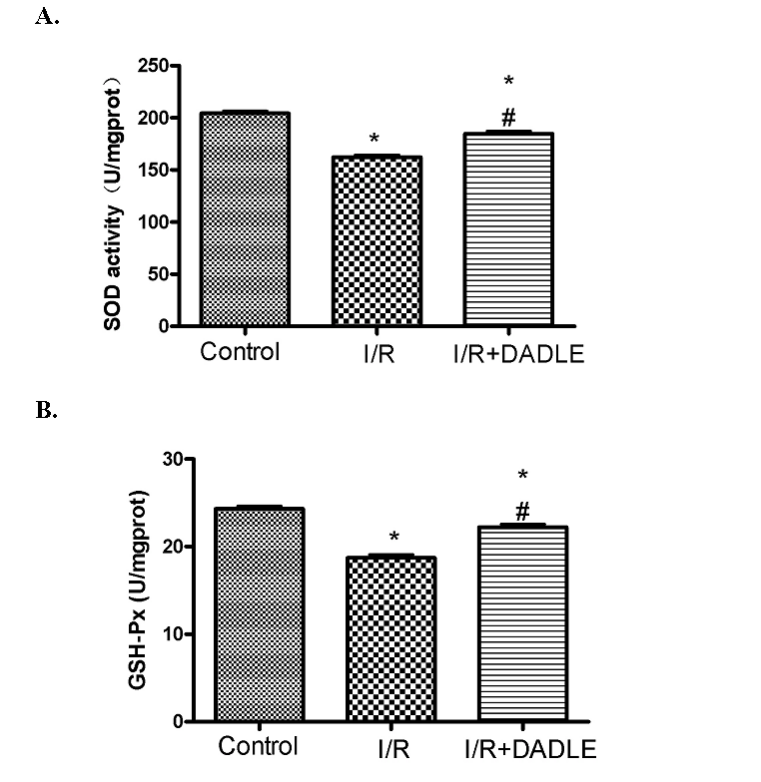
**[D-Ala^2^, D-Leu^5^]-Enkephalinamide (DADLE)-induced increase in superoxide dismutase (SOD) and glutathione peroxidase (GSH-Px)  activities of the cortex exposed to ischemia/reperfusion (I/R) stress**. Mean ± standard deviation (n = 6). * *P *< 0.05 versus the control group. #*P *< 0.05 versus I/R group. Note that I/R induced a significant reduction of SOD and GSH-Px activities, which were increased by **δ**-opioid receptor (DOR) activation with DADLE treatment.

Next, we investigated if DOR activation preserves the antioxidant mechanism because the brain is extremely vulnerable to free radical attack that occurs in ischemia/reperfusion [24,25,30-32]. Since superoxide dismutase (SOD) and glutathione peroxidase (GSH-Px) are the most important enzymes to protect against oxidative stress in the brain [24,25], we measured their activities in the cortex among three groups. As shown in Figure 2B, I/R largely decreased SOD activity from 204.3 ± 5.39 U/mg protein in the control to 162.2 ± 5.91 U/mg protein in the I/R group (*P *< 0.0001), while DOR activation with DADLE significantly increased SOD activity by 22.5 U/mg protein (*P *< 0.0001) in the ischemic cortex. By contrast, GSH-Px activity showed a similar change in response to I/R and DADLE treatment. I/R stress significantly decreased GSH-Px activity by 23% (18.72 ± 0.89 U/mg protein in the I/R group vs 24.31 ± 0.78 U/mg protein in the control, *P *< 0.0001), which was markedly reversed by DADLE (+ 3.50 U/mg protein over the level of the I/R group, *P *< 0.0001) (Figure 2B). These data suggest that I/R stress impairs the antioxidant mechanism, while DOR activation partially restores it.

**Figure 3:**
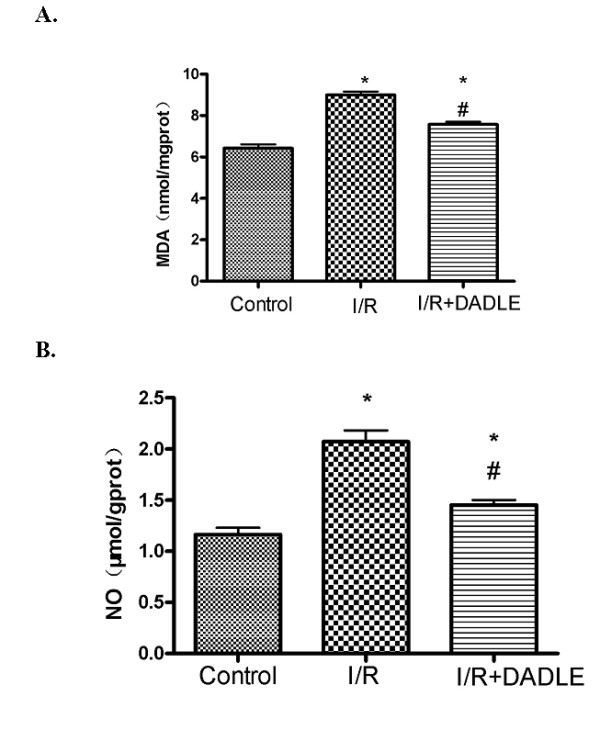
**[D-Ala^2^, D-Leu^5^]-Enkephalinamide (DADLE)-induced decrease in malondialdehyde (MDA) and NO in the cortex exposed to ischemia/reperfusion (I/R) stress**. * *P *< 0.05 versus the control group. #*P *< 0.05 versus I/R group. Note that I/R stress largely increased the contents of MDA and NO in the cortex by 40% to 100%, which was significantly reduced by** δ**-opioid receptor (DOR) activation with DADLE.

Since a reduction of antioxidant enzymes leads to an increase in free radical products[24,25,30-32], especially malondialdehyde (MDA) that damages membrane integrity, we compared the changes in cortical MDA in all groups. The results showed that I/R induced a 40% increase in MDA (8.99 ± 0.41 nmol/mg protein in the I/R group vs. 6.43 ± 0.44 nmol/mg protein in the sham control group, *P *< 0.0001), while DADLE treatment resulted in a significant decrease in the content of MDA (-15.6% vs. I/R alone, *P *< 0.05) (Figure 3A). These results were well consistent with those of antioxidant enzymes, SOD and GSH-Px.

To further ascertain the effect of DOR activation on the antioxidant mechanism, we examined the changes in NO, another critical free radical that causes brain injury in ischemia/reperfusion [24,25]. The data showed that I/R almost doubled the level of NO (2.07 ± 0.27 μmol/g protein in the I/R group vs. 1.16 ± 0.16 μmol/g protein in the control, *P *< 0.0001). This large increase was greatly inhibited by the treatment with DADLE (-29.8%, *P *< 0.0001), lending further support for the DOR-mediated inhibition of free radical production (Figure 3B).

**Figure 4:**
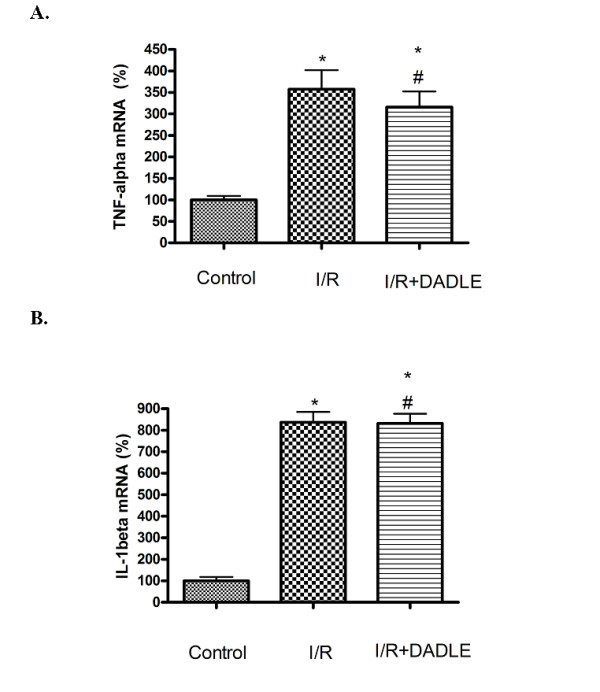
**Effect of [D-Ala^2^, D-Leu^5^]-enkephalinamide (DADLE) on tumor necrosis factor (TNF)α and interleukin (IL)1β mRNA expression of the cortex exposed to ischemia/reperfusion (I/R) stress**. Mean ± standard deviation (n = 6). * *P *< 0.05 versus the control group. #*P *> 0.05 versus I/R group. Note that the cerebral I/R dramatically increased the levels of TNFα and IL1β mRNA by 350% to 700%.

In addition to oxidant free radicals, inflammatory cytokines have been shown to play a role in ischemic injury [25-27]. Therefore, we examined, by quantitative real-time PCR, whether DADLE affects the expression of IL1β and TNFα that are known to be involved in neuronal response to ischemic stress [25-27]. The primer and probe sequences used in this work are presented in Table 1. Our results showed that the levels of both IL1β and TNFα mRNAs tremendously increased after I/R stress (Figure 4). Specifically, IL1β mRNA increased by more than 7-fold (*P *= 0.001 vs. that of the control group) and TNFα mRNA, >2.5-fold (*P *= 0.001 vs that of the control). However, the treatment with DADLE did not induce a significant reduction in ischemic expression of IL1β and TNFα mRNAs at this particular timepoint (72 h after MCAO), suggesting a specific effect of DADLE on antioxidant enzymes and oxidant free radicals shown in Figures 2 and 3.

**Table 1:** Primers and probes

	Sequences of primers and probes
β-Actin	Forward primer: 5'-acggccaggtcactattg-3'
	Reverse primer: 5'-caagaaggaaggctggaaaaga-3'
	Probe: 5'-FAM-caacgagcggttccgatgccct-TAMRA-3'
IL1β	Forward primer: 5'-gatgaaagacggcacacc-3'
	Reverse primer: 5'-ctatgtcccgaccattgc-3'
	Probe: 5'-FAM-aagcggtttgtcttcaacaag-TAMRA-3'
TNFα	Forward primer: 5'-gtgttcatccgttctctaccc-3'
	Reverse primer: 5'-gccacaattccctttctaagtt-3'
	Probe: 5'-FAM-acccctttatcgtctactcctcagagccc-TAMRA-3'

FAM = 6-carboxyfluorescin;

IL = interleukin;

TAMRA = 6-carboxytetramethylrhodamine;

TNF = tumor necrosis factor.

Since our previous work has suggested that ERK activation is neuroprotective and may mediate the DOR-induced neuroprotection in cortical neurons [6], we further investigated if it is involved in the DOR-induced protection from I/R injury. Therefore, we proposed to determine the effect of ERK kinase inhibition on the ischemic brain by applying PD98059, an ERK inhibitor, into the lateral ventricle of the brain. Unfortunately, most of the animals (10 out of 12 rats), either with or without DADLE, died before the timepoint (72 h after MCAO) set for biochemical or molecular measurements. The death rate (>83%) was significantly higher than that of all other groups. This observation suggests that the inhibition of ERK kinase greatly exacerbated the I/R injury and increased animal death rate.

## Discussion

We have made two major findings in the cortex exposed to cerebral ischemia and reperfusion: (1) DOR activation partially reversed the I/R-induced decrease in SOD and GSH-Px activity and (2) DOR activation decreased the content of MDA and NO, suggesting that DOR activation attenuates oxidative injury in the I/R brain. In addition, we observed that the inhibitor of ERK kinase that mediates DOR neuroprotection [6] accelerated the death of the I/R animals, while DOR activation reduced the production of caspase 3 that triggers neuronal death [25,28,29].

This is the first study showing that DOR activation increases SOD and GSH-Px activity in the brain exposed to ischemia/reperfusion. Neuronal function is dependent on the integrated dynamics of the membrane lipid matrix, which is vulnerable to free radical attack due to ischemic/oxidative stress [24,25,30-32]. Free radicals are compounds possessing an unpaired electron, which renders them highly reactive and capable of causing oxidative damage to all the major macromolecules, including lipids, proteins and nucleic acids in cells. A major family of free radicals is the reactive oxygen species (ROS) and another important free radical is NO [25,30-34]. In normal circumstances, the brain is protected from free radical attack by antioxidant mechanisms that include antioxidant enzymes (for example, SOD and GSH-Px) and free radical scavenging substances (for example, ascorbate, vitamin E and protein sulfhydryls) [24,25,30-34]. Lack of oxygen and/or blood supply in relation to aerobic ATP requirements and oxygen/blood reperfusion cause an increase in the generation of ROS and NO. Oxidative injury occurs when the production of free radicals is greater than the ability of the cell to repair the resulting damage [24,25,30-33]. Ischemic/hypoxic injury may be intensified by concurrent oxidative injury. Polyunsaturated fatty acids (PUFAs) are among the molecules most susceptible to ROS. The oxidative breakdown of membrane PUFAs yields toxic MDA, which compromises not only membrane lipid matrix dynamics, and hence structure and function of membrane-associated proteins such as enzymes, receptors, and transporters, but also gene expression [24,25,32]. By contrast, excessive NO leads to the release of cytochrome *c *from the mitochondria and triggers cellular death through caspase pathway [25,33]. Indeed, we observed a large decrease in antioxidant enzymes with a marked increase in MDA and NO in the ischemic brain. Such changes could injure the brain without doubt. Interestingly, our present data suggest that these pathophysiological changes could be partially reversed by DOR activation. This finding may provide a clue for new strategies against hypoxic/ischemic brain injury.

Caspases are synthesized in most cells, including neurons, as inactive precursors, with caspase 3 being a terminal enzyme in the caspase family that activates an endonuclease (caspase-activated DNAse), resulting in DNA fragmentation and neuronal death in ischemia [25,28,29]. Since DOR activation inhibits the production of NO in the ischemic brain, it was not a surprise to see a decrease in caspase 3 expression in our study. NO, a water and lipid-soluble free radical, is an inorganic gas that has an extremely short half-life. This unstable nitrogen radical plays an important role in the control of cerebral blood flow and thrombogenesis and modulation of neuronal activity. However, I/R stress may stimulate NO synthase and result in excessive amounts of NO in the brain. Excessive production of NO causes cytochrome *c *release from the mitochondria and activates caspase signal pathway, thus triggering neuronal death through the caspase-activated DNase activation [25,34]. In our previous study, we have observed that DOR activation attenuated cytochrome *c *release in severe hypoxic stress [6]. Taken together with the present results, it is likely that DOR activation may reduce the production of NO, attenuate cytochrome *c *release and decrease caspase activity, thus protecting the brain from ischemia/reperfusion injury.

In our previous exploration of the mechanism underlying DOR neuroprotection, we showed that ERK is involved in the DOR signaling in neuroprotection [6]. Subsequently, another independent laboratory also demonstrated that ERK signaling plays an important role in DOR-mediated neuroprotection [17]. However, both our and other studies were conducted in *in vitro *models [6,17]. It is unclear whether ERK mediates DOR neuroprotection in the *in vivo *model, especially in the ischemic brain. We therefore proposed to address this issue in this work. Unfortunately, most animals died after microinjection of ERK inhibitor into the ventricle, even with DADLE treatment. This jeopardized our further studies on these animals. However, such an outcome does provide hints implying that ERK, consistent with our previous finding [6], is in a key position in an intracellular pathway mediating DOR neuroprotection because (1) the blockade of ERK exacerbated ischemia/reperfusion injury and (2) DOR activation could not protect the animals from ischemia/reperfusion injury after the blockade of ERK in the brain.

The data on IL1β and TNFα mRNA expression suggest that the effect of DOR activation on oxidative injury is specific in this work. However, we cannot rule out the possibility that DOR signals may target proinflammatory cytokines after I/R stress since many cellular and molecular mechanisms function differently at various stages of pathophysiological events. It is essential to perform more studies at different timepoints along the course of such events to draw solid conclusions as to whether DOR activation has any effect on inflammatory response to ischemia/reperfusion.

## Conclusion

**Figure 5:**
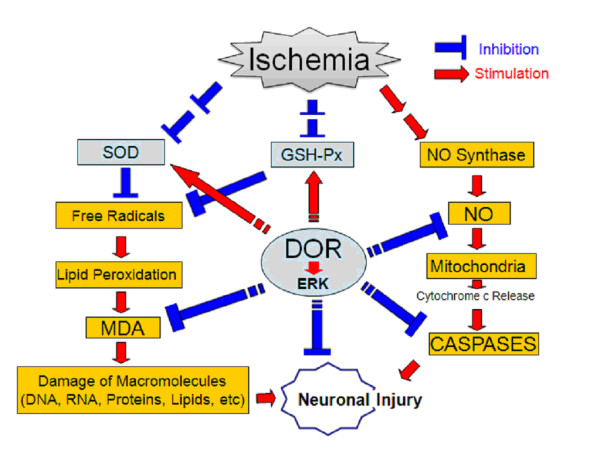
**Schematic diagram showing potential mechanism of δ-opioid receptor (DOR) protection from oxidative injury in the brain exposed ischemia/reperfusion**.

We have elucidated the mechanism of DOR protection from cerebral ischemia by investigating the effect of DOR activation on antioxidant mechanisms in the brain exposed to ischemia and reperfusion. Our data show that DOR activation greatly enhances the activity of antioxidant enzymes and reduces free radicals, MDA and NO, in the brain after cerebral ischemia and reperfusion. The results suggest that DOR activation attenuates oxidative injury, which may be one of the important mechanisms underlying DOR protection from ischemic injury [5,12,16,18-21]. Taken together with our previous studies, the potential mechanism of DOR neuroprotection from I/R injury is schematically demonstrated (Figure 5).

## Methods

### Animals

Adult male Sprague-Dawley rats weighing 250 to 270 g were purchased from the Experimental Animal Center of Soochow University (Suzhou, Jiangsu, China). Animals were housed in a room with temperature of 21 to 23°C, relative humidity at 30% to 70%, and a 12-h light/12-h dark cycle. They had free access to food and water. All experimental procedures in this study were performed in accordance with the guidelines for the care and use of laboratory animals of Soochow University.

### Experimental groups

The rats, under deep anesthesia, were exposed to cerebral ischemia (see below) for 2 h followed by 72-h reperfusion to induce I/R injury in the brain. We have previously shown DOR activation induced protection from ischemic injury [5,12]. Since this study was mainly for the purpose of determining if DOR activation has any effect on major antioxidant enzymes and oxidant free radicals after I/R, we randomly assigned the animals to various groups after the same procedure for cerebral ischemia (see below), and sampled cortical and hippocampal tissues for biochemical and molecular measurements. The animals that died before the timepoint (72 h after MACO) were removed from the experiments, since no brain tissues could be sampled from them. There was no significant change in the death rate among various groups except for the PD98059 group (see below and Results).

In the I/R plus DOR activation group (n = 12), the I/R rats were treated with DADLE, a DOR agonist purchased from Sigma (St Louis, MO, USA). DADLE was applied through intraperitoneal injection (1 mg/kg in saline) [18] within 30 min starting at 30 min after the onset of suture occlusion. The reason we chose to apply DADLE in the early stage of ischemia was because an early application of DADLE displays better neuroprotection against excitotoxic injury, as shown in our previous work [3]. In the I/R control group (n = 12), the procedures were the same except for vehicle solution without DADLE. In the sham control group (n = 6), rats were subjected to sham I/R operation. In addition, in a group of rats (n = 12), PD98059 (Sigma, St Louis, MO, USA; 1 μg in 6 μl of vehicle solution), a specific inhibitor of mitogen-activated protein kinase (MAPK) extracellular signaling-regulated kinase (ERK) kinase, was microinjected into the right lateral ventricle of the ischemic rats with or without DADLE, through a stereotaxically inserted 27-gauge needle (ALCB10, Shanghai Alcott Biotech, Shanghai, China) (1.5 mm lateral and 1 mm posterior to the bregma, 4 mm in depth) [35]. The microinjection was started at 30 min after the onset of cerebral ischemia and was completed within 30 min. After the microinjection, sterile procedures were performed to suture the skin.

### Induction of cerebral ischemia

To mimic cerebral I/R injury, the model of stroke was established by performing MCAO in the rat [5,12,36]. In brief, rectal temperature was recorded and maintained at 37°C ± 0.5°C throughout the surgical procedure and up to 2 h after reperfusion. The right common carotid artery was carefully separated from around tissues and a 4-0 monofilament nylon suture coated with paraffin was inserted through the external carotid artery stump into the internal carotid artery to occlude the right middle cerebral artery. After 2 h of occlusion, the nylon monofilament suture was withdrawn for blood flow reperfusion. In the sham-operated group, the surgical procedures were the same as in the ischemic animals except for real insertion of the suture.

### Sample preparation

After 72-h reperfusion, the rat brains were quickly removed under deep anesthesia and placed into cold ethylenediaminetetraacetic acid (EDTA) buffer (0.02 M, pH = 7.4) for removing tissue debris and blood clots. All visible vascular structures were also removed. Then, the cortex and hippocampus were dissected from the hemispheres, respectively.

For immunohistochemistry, the tissues were fixed in 10% formalin, pH 7.4. After dehydration in graded ethanol and xylene, the tissues were paraffin embedded and cut into coronal sections (4 μm thick). For RNA and other measurements, the samples were frozen in liquid nitrogen and stored at -80°C until use.

For detection of the activities of antioxidant enzymes (SOD and GSH-Px) and the content of MDA and nitric oxide (NO), the tissue samples were weighed and homogenized with a buffer consisting of 10 mM sucrose, 10 mM tris(hydroxymethyl)aminomethane (Tris)-HCl, and 0.1 mM EDTA (pH 7.4), and then centrifuged at 3,000 *g *for 15 min at 4°C. The supernatant was used for bioassays. Total protein concentration was measured using the method of bicinchoninic acid protein assay. All the data were normalized to protein concentration (a unit value per μg of protein).

### Caspase 3 immunohistochemistry

Procedures were processed according to the manufacturer's protocol recommended for the caspase 3 immunohistochemistry kit (Beijing Zhongshan Biotechnology, Beijing, China). Following deparaffinzation and rehydration, brain sections were irradiated in 0.1 mol/l sodium citrate buffer (pH 6.0) in a microwave oven for 12 min. The sections were then exposed to 3% H_2_O_2 _for 10 min to bleach endogenous peroxidases, and subsequently subjected to three rinses with phosphate-buffered saline (PBS) for 10 min each. These sections were incubated with an anti-caspase 3 primary antibody (1:100, Beijing Zhongshan Biotechnology) for 1 h at 37°C and then washed in PBS 3 times. Finally, the sections were incubated with a biotinylated goat secondary anti-mouse antibody (Beijing Zhongshan Biotechnology) for 30 min at 37°C. The primary antibody was omitted in the control sections. After washing in PBS, caspase 3 signals were visualized with 3,3'-diaminobenzidine tetrahydrochloride (DAB) and counterstained with hematoxyline. Finally, the sections were dehydrated in graded ethanol, immersed in xylene and covered by cover slips.

The number of caspase 3-positive cells was blindly counted by a researcher blinded to the assignment of experimental groups. In brief, images were taken with an Olympus BX-51 microscope (Olympus, Tokyo, Japan). Cells with a blue color were counted as neurons. Cells showing both blue and brown color were counted as caspase 3-positive neurons. At least three view areas were randomly selected for a given region on each section. The ratio of caspase 3-positive cells per neurons in each view area was calculated and then three or more values from the randomly selected view areas were averaged to represent the value of the region.

### Assay of SOD activity

SOD activity was measured using an assay kit from Nanjing Jiancheng Bioengineering Institute (Nanjing, China) according to the manufacturer's protocol. It uses a xanthine-xanthine oxidase system to determine the inhibition of nitroblue tetrazolium (NBT) reduction due to superoxide anion generation. Briefly, 500 μl of supernatant was mixed with 50 μM xanthine and 2.5 μM xanthine oxidase in 50 μM potassium phosphate buffer and incubated at 37°C for 40 min. Then, NBT is added. Total nitrite (nitrite + nitrate) produced by the oxidation of oxyamine was measured by detecting the absorbance at 550 nm. A unit of SOD was defined as the enzyme amount causing 50% inhibition of the NBT reduction rate. The SOD activity was expressed as units of nitrite per mg protein.

### Assay of GSH-Px activity

The GSH-Px activity was determined using an assay kit from Nanjing Jiancheng Bioengineering Institute according to the manufacturer's protocol. In brief, 200 μl of the supernatant was mixed with 2.68 ml of 0.05 M phosphate buffer (pH 7.0) containing 5 mM of EDTA, followed by the addition of 0.1 ml of 8.4 mM nicotinamide adenine dinucleotide phosphate (NADPH), 0.01 ml of glutathione reductase, 0.01 ml of 1.125 M NaN_2_, and 0.1 ml of 0.15 M glutathione. The enzymatic reaction was initiated by the addition of 0.1 ml of 2.2-mM H_2_O_2_. The changes in absorbance at 340 nm were continuously recorded between 2 and 4 min after initiation of the reaction (converting NADPH to NADP). A GSH-Px unit was defined as the enzyme activity required to convert 1 mmol of NADPH to NADP per mg tissue protein. The GSH-Px activity was expressed as units per mg protein.

### MDA assay

The MDA content was determined by the thiobarbituric acid method using an assay kit from Nanjing Jiancheng Bioengineering Institute according to the manufacturer's recommendations. It was assayed in the form of thiobarbituric acid reacting substances (TBARS). A total of 100 μl of the supernatant was mixed with 1 ml of 20% trichloroacetic acid and 1.0 ml of 0.1% TBARS reagent and incubated at 95°C for 80 min. After cooling on ice, the mixture was centrifuged at 1,000 *g *for 20 min. The absorbance of the supernatant was measured at 532 nm. The amount of TBARS was determined using tetraethoxypropane as a standard. The content of TBARS, as an index of MDA, was expressed as nmol per mg protein.

### Nitric oxide assay

The concentration of nitrite was measured to reflect the production of NO using a Griess reagent system kit (Jiancheng Institute of Biotechnology), according to the manufacturer's recommendations. In brief, the supernatant was mixed with the Griess reagent (1% sulfanilamide, 0.1% *N*-l-naphathyletylenediamine dihydrochloride and 2.5% phosphoric acid) at room temperature for 10 min. Nitrite products in the supernatants were determined by measuring absorbance at 550 nm with NaNO_2 _being used for a standard curve. The results were expressed as μmol/g protein.

### Quantitative real-time polymerase chain reaction (PCR)

Total RNA was extracted from the brain tissues with Trizol reagent (Invitrogen, Carlsbad, CA, USA) according to the manufacturer's protocol and then reverse transcribed using a RevertAid First Strand cDNA Synthesis kit (Fermentas, Vilnius, Lithuania).

The cDNA sequences for IL1β, TNFα, and β-actin were obtained from NCBI GenBank. Specific PCR primers and TaqMan fluorogenic probes were designed using Primer Express software (Applied Biosystems, Foster City, CA, USA) and commercially synthesized by Invitrogen. 6-Carboxyfluorescin (6-FAM) was used as the reporter dye and 6-carboxytetramethylrhodamine (TAMRA) as the quencher dye.

Real-time reverse transcriptase (RT)-PCR was performed with TaqMan Universal PCR Master Mix (Applied Biosystems) on a Lightcycle (Roche, Basel, Switzerland). Briefly, the reaction mixture contained cDNA 2 μl, 10 × buffer 2.5 μl, 25 mM MgCl_2 _1.5 μl, 10 mM dNTP 0.5 μl, Taq 0.5 μl, 0.1 μl each of the 100 μM sense and antisense primers and 0.1 μl of 100 μM probe. All samples were tested in duplicate. The conditions of amplification for TNFα were 95°C for 2.5 min, 95°C for 5 s and 56°C for 45 s for 40 cycles. The conditions of amplification for IL1β were 95°C for 2.5 min, 95°C for 15 s and 60°C for 45 s for 40 cycles. The amplification for β-actin was performed according to the manufacturer's instructions (Invitrogen).

The relative levels of these mRNAs were calculated by 2^-ΔΔCT ^method and the levels of TNFα and IL1β mRNAs were normalized to that of β-actin.

### Statistical analysis

All data were subjected to statistical analysis using Graph Prism v. 5.0 software (GraphPad Software, San Diego, CA, USA). Statistical significances among groups were determined by one-way analysis of variance (ANOVA) or unpaired t test. *P *< 0.05 was considered as statistically significant.

## Authors' contributions

YLY participated in the design of the study, performed experiments, and helped draft the manuscript. XWX performed experiments. YZ and QW participated in the design of the study, performed experiments and statistical analysis, and helped draft the manuscript. LL performed partial experiments. GHL participated in the design of the study and performed partial experiments. YX participated in the design of the study, interpreted the results and wrote the manuscript. All authors have read and approved the manuscript.

## References

[B1] Higgins GC, Beart PM, Nagley P (2009). Oxidative stress triggers neuronal caspase-independent death: endonuclease G involvement in programmed cell death-type III. Cell Mol Life Sci.

[B2] Locatelli F, Ballabio E, Lanfranconi S, Papadimitriou D, Strazzer S, Bresolin N, Comi GP, Corti S (2009). Stem cell therapy in stroke. Cell Mol Life Sci.

[B3] Zhang JH, Xia Y, Haddad GG (1999). Activation of δ-opioid receptors protects cortical neurons from glutamate excitotoxic injury. Soc Neurosci.

[B4] Zhang JH, Gibney GT, Zhao P, Xia Y (2002). Neuroprotective role of δ-opioid receptors in the cortical neurons. Am J Physiol.

[B5] Zhao P, Guo JC, Xia Y, Hong SS, Bazzy-Assad A, Cheng JS, Xia Y (2002). Electro-acupuncture and brain protection from cerebral ischemia: The role of δ-opioid receptor. Soc Neurosci.

[B6] Ma MC, Qian H, Ghassemi F, Zhao P, Xia Y (2005). Oxygen sensitive δ-opioid receptor-regulated survival and death signals: novel insights into neuronal preconditioning and protection. J Biol Chem.

[B7] Zhang J, Qian H, Zhao P, Hong SS, Xia Y (2006). Rapid hypoxia preconditioning protects cortical neurons from glutamate toxicity through δ-opioid receptor. Stroke.

[B8] Chao D, Donnelly DF, Feng Y, Bazzy-Asaad A, Xia Y (2007). Cortical δ-opioid receptors potentiate K^+ ^homeostasis during anoxia and oxygen-glucose deprivation. J Cereb Blood Flow Metab.

[B9] Chao D, Bazzy-Asaad A, Balboni G, Xia Y (2007). δ-, but not μ-, opioid receptor stabilizes K^+ ^homeostasis by reducing Ca^2+ ^influx in the cortex during acute hypoxia. J Cell Physiol.

[B10] Chao D, Bazzy-Asaad A, Balboni G, Salvadori S, Xia Y (2008). Activation of DOR attenuates anoxic K^+ ^derangement via inhibition of Na^+ ^entry in mouse cortex. Cereb Cortex.

[B11] Hong SS, Qian H, Zhao P, Bazzy-Asaad A, Xia Y (2007). Anisomycin protects cortical neurons from prolonged hypoxia with differential regulation of p38 and ERK. Brain Res.

[B12] Tian XS, Zhou F, Yang R, Xia Y, Wu GC, Guo JC (2008). Effects of intracerebroventricular injection of δ-opioid receptor agonist TAN-67 or antagonist naltrindole on acute cerebral ischemia in rats. Acta Physiologica Sinica.

[B13] Kang XZ, Chao DM, Gu QB, Ding GH, Wang YW, Balboni G, Lazarus LH, Xia Y (2009). δ-Opioid receptors protect from anoxic disruption of Na^+ ^homeostasis via Na^+ ^channel regulation. Cell Mol Life Sci.

[B14] Borlongan CV, Wang Y, Su TP (2004). δ Opioid peptide (D-Ala 2, D-Leu 5) enkephalin: linking hibernation and neuroprotection. Front Biosci.

[B15] Borlongan CV, Su TP, Wang Y (2001). δ Opioid peptide augments functional effects and intrastriatal graft survival of rat fetal ventral mesencephalic cells. Cell Transplant.

[B16] Borlongan CV, Hayashi T, Oeltgen PR, Su TP, Wang Y (2009). Hibernation-like state induced by an opioid peptide protects against experimental stroke. BMC Biol.

[B17] Narita M, Kuzumaki N, Miyatake M, Sato F, Wachi H, Seyama Y, Suzuki T (2006). Role of δ-opioid receptor function in neurogenesis and neuroprotection. J Neurochem.

[B18] Iwata M, Inoue S, Kawaguchi M, Nakamura M, Konishi N, Furuya H (2007). Effects of δ-opioid receptor stimulation and inhibition on hippocampal survival in a rat model of forebrain ischaemia. Br J Anaesth.

[B19] Su DS, Wang ZH, Zheng YJ, Zhao YH, Wang XR (2007). Dose-dependent neuroprotection of δ opioid peptide [D-Ala2, D-Leu5] enkephalin in neuronal death and retarded behavior induced by forebrain ischemia in rats. Neurosci Lett.

[B20] Xiong LZ, Yang J, Wang Q, Lu ZH (2007). Involvement of δ- and μ-opioid receptors in the delayed cerebral ischemic tolerance induced by repeated electroacupuncture preconditioning in rats. Chin Med J.

[B21] Charron C, Messier C, Plamondon H (2008). Neuroprotection and functional recovery conferred by administration of kappa- and δ-opioid agonists in a rat model of global ischemia. Physiol Behav.

[B22] Pamenter ME, Buck LT (2008). δ-Opioid receptor antagonism induces NMDA receptor-dependent excitotoxicity in anoxic turtle cortex. J Exp Biol.

[B23] Horiuchi T, Kawaguchi M, Kurita N, Inoue S, Sakamoto T, Nakamura M, Konishi N, Furuya H (2008). Effects of δ-opioid agonist SNC80 on white matter injury following spinal cord ischemia in normothermic and mildly hypothermic rats. J Anesth.

[B24] Behn C, Araneda OF, Llanos AJ, Celedón G, González G (2007). Hypoxia-related lipid peroxidation: evidences, implications and approaches. Respir Physiol Neurobiol.

[B25] Sung JH, Chao DM, Xia Y, Wang Q, Ying WH (2008). Neuronal responses to hypoxia. New Frontiers in Neurological Research.

[B26] Huang J, Upadhyay UM, Tamargo RJ (2006). Inflammation in stroke and focal cerebral ischemia. Surg Neurol.

[B27] Adibhatla RM, Hatcher JF (2007). Secretory phospholipase A(2) IIA is up-regulated by TNF-α and IL-1α/β after transient focal cerebral ischemia in rat. Brain Res.

[B28] Namura S, Zhu J, Fink K, Endres M, Srinivasan A, Tomaselli KJ, Yuan J, Moskowitz MA (1998). Activation and cleavage of caspase-3 in apoptosis induced by experimental cerebral ischemia. J Neurosci.

[B29] Zhang H, Gao X, Yan Z, Ren C, Shimohata T, Steinberg GK, Zhao H (2008). Inhibiting caspase-3 activity blocks β-catenin degradation after focal ischemia in rat. Neuroreport.

[B30] Fujimura M, Tominaga T, Chan PH (2005). europrotective effect of an antioxidant in ischemic brain injury: involvement of neuronal apoptosis. Neurocrit Care.

[B31] Lievre V, Becuwe P, Bianchi A, Bossenmeyer-Pourie C, Koziel V, Franck P, Nicolas MB, Dauca M, Vert P, Daval JL (2001). Intracellular generation of free radicals and modifications of detoxifying enzymes in cultured neurons from the developing rat forebrain in response to transient hypoxia. Neuroscience.

[B32] Potashkin JA, Meredith GE (2006). The role of oxidative stress in the dysregulation of gene expression and protein metabolism in neurodegenerative disease. Antioxid Redox Signal.

[B33] Dezfulian C, Raat N, Shiva S, Gladwin MT (2007). Role of the anion nitrite in ischemia-reperfusion cytoprotection and therapeutics. Cardiovasc Res.

[B34] Ryter SW, Kim HP, Hoetzel A, Park JW, Nakahira K, Wang X, Choi AM (2007). Mechanisms of cell death in oxidative stress. Antioxid Redox Signal.

[B35] Bures J, Petrin M, Zachar J (1960). Electrophysiological methods in biological research.

[B36] Longa EZ, Weinstein PR, Carlson S, Cummins R (1989). Reversible middle cerebral artery occlusion without craniectomy in rats. Stroke.

